# Trajectories of Early Childhood Developmental Skills and Early Adolescent Psychotic Experiences: Findings from the ALSPAC UK Birth Cohort

**DOI:** 10.3389/fpsyg.2017.02314

**Published:** 2018-01-09

**Authors:** Mohajer A. Hameed, Raghu Lingam, Stanley Zammit, Giovanni Salvi, Sarah Sullivan, Andrew J. Lewis

**Affiliations:** ^1^School of Psychology, Faculty of Health, Deakin University, Melbourne, VIC, Australia; ^2^Institute of Health and Society, Newcastle University, Newcastle upon Tyne, United Kingdom; ^3^Centre for Academic Mental Health, University of Bristol, Bristol, United Kingdom; ^4^MRC Centre for Neuropsychiatric Genetics and Genomics, Cardiff University, Cardiff, United Kingdom; ^5^Devon Partnership NHS Trust, Exeter, United Kingdom; ^6^NIHR CLAHRC West, Bristol, United Kingdom; ^7^School of Psychology and Exercise Science, Murdoch University, Perth, WA, Australia

**Keywords:** trajectories of early childhood development, psychotic experiences, adolescence, ALSPAC birth cohort

## Abstract

**Objective:** The aim of this study was to use prospective data from the Avon Longitudinal Study of Parents and Children (ALSPAC) to examine association between trajectories of early childhood developmental skills and psychotic experiences (PEs) in early adolescence.

**Method:** This study examined data from *n* = 6790 children from the ALSPAC cohort who participated in a semi-structured interview to assess PEs at age 12. Child development was measured using parental report at 6, 18, 30, and 42 months of age using a questionnaire of items adapted from the Denver Developmental Screening Test – II. Latent class growth analysis was used to generate trajectories over time for measures of fine and gross motor development, social, and communication skills. Logistic regression was used to investigate associations between developmental trajectories in each of these early developmental domains and PEs at age 12.

**Results:** The results provided evidence that decline rather than enduringly poor social (adjusted OR = 1.28, 95% CI = 1.10–1.92, *p* = 0.044) and communication skills (adjusted OR 1.12, 95% CI = 1.03–1.22, *p* = 0.010) is predictive of suspected or definite PEs in early adolescence, than those with stable and/or improving skills. Motor skills did not display the same pattern of association; although gender specific effects provided evidence that only declining pattern of fine motor skills was associated with suspected and definite PEs in males compared to females (interaction OR = 1.47, 95% CI = 1.09–1.97, *p* = 0.012).

**Conclusion:** Findings suggest that decline rather than persistent impairment in social and communication skills were most predictive of PEs in early adolescence. Findings are discussed in terms of study’s strengths, limitations, and clinical implications.

## Introduction

Over the last decade, there has been increasing interest in early adolescent psychotic experiences (PEs) in the general population as a sign of vulnerability to adult psychopathologies including schizophrenia-spectrum disorders (Nuevo et al., 2012; Hameed, 2013). While a number of prospective cohort studies have examined various aspects of early childhood developmental abnormalities as predictors of adult onset schizophrenia-spectrum disorders, findings have been equivocal, in particular in relation to change in developmental abilities over time (Jones et al., 1994; Erlenmeyer-Kimling et al., 2000; Rosso et al., 2000; Cannon et al., 2002; Owens and Johnstone, 2006; Welham et al., 2009a). This paper draws on data from a population cohort study to examine trajectories of early childhood developmental skills and PEs in early adolescence.

A systematic review and meta-analysis of population-based studies of PEs in childhood and adolescence reported that the median prevalence among children aged 9–12 years was higher (17%) than adolescents aged 13–18 years (7.5%) (Kelleher et al., 2012). Accumulated evidence indicate that PEs are relatively common in early adolescence (van Os et al., 2000; Dhossche et al., 2002; Rossler et al., 2007; Horwood et al., 2008; Kelleher and Cannon, 2011; Laurens et al., 2012). However, these experiences (e.g., hallucinations, delusions, and thought broadcasting) do not represent a clinical diagnosis and are not, in themselves, an indication for clinical intervention. Furthermore, presence of PEs in early adolescence do not necessarily suggest a developmental trajectory toward severe psychopathology and/or functional impairment. However, the accumulated evidence suggests that while PEs in early adolescence are not specific to a diagnosis of any schizophrenia-spectrum disorders in adulthood, these symptoms are useful markers and indicators of adult mental health problems more broadly (Poulton et al., 2000; Fisher et al., 2013). In particular, PEs have been associated with distress and often co-morbid with depressive symptoms in a community sample of adolescents and young adults (Armando et al., 2010). PEs in adolescence can therefore be conceptualized as a non-specific developmental predictor of a broad range of adult mental health problems (Nuevo et al., 2012; Fisher et al., 2013; Zammit et al., 2013).

Previous prospective studies have investigated developmental domains such as child literacy (Hameed et al., 2013), social cognition (Thompson et al., 2011a; Jones et al., 2012), intellectual profile (Horwood et al., 2008), cognitive styles (Thompson et al., 2011b; Sullivan et al., 2013a), peer relationship (Schreier et al., 2009) and risk of PEs in early adolescence. Similarly, the emerging body of prospective studies have examined the association of early childhood developmental skills and risk of adult onset schizophrenia-spectrum disorders. A systematic review of longitudinal Familial High Risk (FHR; children of parents presenting with schizophrenia) examined the developmental features across childhood as predictors of adult onset schizophrenia (Hameed and Lewis, 2016). Several longitudinal FHR studies have found that children who later developed schizophrenia-spectrum disorders showed impaired early developmental skills (e.g., fine, gross motor skills, motor coordination and balance, psychomotor development, verbal abilities) (Erlenmeyer-Kimling and Cornblatt, 1987; Fish, 1987; Marcus et al., 1987; McNeil and Kaij, 1987; Fish et al., 1992), consistent with the literature on developmental precursors of schizophrenia-spectrum disorders (Walker et al., 1994; Schiffman et al., 2004).

A number of findings indicate that children who later develop schizophrenia-spectrum disorders show differences in multiple developmental domains of functioning well before the onset of psychotic symptoms. For example, studies using data from the Northern Finland 1966 Birth Cohort (Isohanni et al., 2001), British 1946 birth cohort (Jones et al., 1994), Dunedin Multidisciplinary Health and Development Study (Cannon et al., 2002), have consistently found that later attainment of developmental milestones such as age at learning to stand, walk and becoming toilet-trained, were related to an increased risk of subsequent schizophrenia-spectrum disorders compared to normal or early attainment.

However, there are relatively few studies that have examined change in early childhood developmental skills as a risk factor for later schizophrenia-spectrum disorders or psychotic symptoms in early adolescence. A review of birth cohort studies of schizophrenia-spectrum disorders (Welham et al., 2009a), found only five studies (Done et al., 1994; Jones et al., 1994; Crow et al., 1995; Bearden et al., 2000; Welham et al., 2009b) that examined change in childhood behavior over time, and additional three studies (Kremen et al., 1998; Cannon et al., 2000; Osler et al., 2007) investigated declining patterns in IQ and risk of schizophrenia-spectrum disorders in adulthood. Welham et al. (2009a) indicated that the majority of the reviewed studies used mean group differences in behavior change and cognitive abilities over time (e.g., declining patterns in cognitive skills acquisition), rather than within-individual change measures; the former may pose limitations to data analysis.

To combat this limitation, this study utilize trajectory modeling to examine patterns of early development and skill acquisition as predictors of later PEs. Therefore, this study adopts a person-oriented approach to child development (Sterba and Bauer, 2010), which assumes that there are meaningful subgroups of individuals who have common pathways of development. In contrast to previous work, we will use a within-individual change measures analysis rather than considering the mean group differences; this is considered the most robust method to analyze developmental change over time. Heterogeneity of developmental trajectories (or distinct classes) may exist within the larger population (Jung and Wickrama, 2007). For example, acquisition of developmental skills such as motor milestones is more likely characterized by individual variations in development at each time point and over time, and timing relative to acquisition of other milestones (Atun-Einy et al., 2012).

To our knowledge, this is the first study to examine the association between trajectories of early childhood developmental skills and PEs in early adolescence. The aim of this investigation is to use prospective data to investigate the association between early childhood developmental skills acquisition across the first 4 years of life (e.g., fine, gross motor, social, and communication skills) and risk of PEs assessed at 12 years of age. It was hypothesized that relative to those with a stable and improving trend of performance, those with deteriorating developmental scores over time would have increased risk of PEs.

## Materials and Methods

### Sample

This study examined longitudinal data from the Avon Longitudinal Study of Parents and Children (ALSPAC). The ALSPAC cohort profile has been described previously (Boyd et al., 2012; Fraser et al., 2012). Parents have completed regular postal questionnaires about various aspects of their child’s health and development since birth. The children have attended annual assessment clinics since age 7 years (Boyd et al., 2012; Fraser et al., 2012). However, due to attrition and non-response, the sample sizes in this study differs according to the time point of data examined. Proposal and ethical approval for the study was obtained from the ALSPAC’s Law and Ethics Committee and the Deakin University Human Research Ethics Committee. Written informed consent was obtained from the participants after explanation of the nature of the study. The research study adheres to the tenets of the Declaration of Helsinki (Horwood et al., 2008).

### Dataset

This study examined data from *N* = 6790 (44.6%) children from the ALSPAC cohort who participated in the psychosis-like symptoms (PLIKS) interview at mean age 12.9 (range = 12.5–13.3 years).

### Measures

#### Psychotic Experiences (PEs) Interview at 12 Years

Psychotic experiences were measured at an ALSPAC clinic using the psychosis-like symptoms (PLIKS) semi-structured face-to-face interview (Horwood et al., 2008), which consisted of 12 core items enquiring about experience of hallucinations (visual and auditory); delusions (delusions of being spied on, persecution, thoughts being read, reference, control, grandiose ability, and other unspecified delusions); and experiences of thought interference (thought broadcasting, insertion, and withdrawal) over the previous 6 months. For these core items, seven stem questions were derived from the Diagnostic Interview Schedule for Children-IV (DISC-IV) (Shaffer et al., 2000) and five from sections 17–19 of the Schedules for Clinical Assessment in Neuropsychiatry version 2.0 (SCAN 2.0) (WHO, 1994), modified slightly after piloting. Psychology graduates trained in the assessment of the SCAN psychosis section read out a stem question (e.g., since your 12th birthday, have you ever heard voices that other people can’t hear?) from the interview schedule and then presented a card with “yes, no, or maybe” response. The semi-structured part of the interview then enabled probing for more information and clarification of answers, ensuring valid data collection.

The coding of all items followed the glossary definitions, assessment, and rating rules for the SCAN. The items were rated as *not present, suspected, or definitely present* with an average *κ*-value for inter-rater reliability of 0.72 (Horwood et al., 2008). Present symptoms were only included if these were not attributable to sleep, fever or substance use. Our primary outcome was presence of either suspected or definite (PEs combined group). As secondary analyses we also explored whether associations were stronger for definite as opposed to suspected PEs. In this study, PEs were classified as not present (*N* = 5862, 86.3%), suspected (*N* = 544, 8.0%), and definitely present (*N* = 384, 5.7%).

#### Early Childhood Developmental Skills

Child development was measured from maternal report using a questionnaire incorporating items adapted from the Denver Developmental Screening Test – II (DDST – II) (Frankenburg and Dodds, 1967), a developmental screening tool used to assess whether children reach age-appropriate milestones. The items selected were those shown to be most predictive of developmental issues and were adapted for research after piloting and discussion with focus groups. The scale has been widely used in ALSPAC and comparison with the Griffiths Developmental Scale, administered to a representative subpopulation of the cohort (*n* = 1045) at 18 months, showed a correlation coefficient of 0⋅54 (*p* < 0.0001) (Hibbeln et al., 2007; Joinson et al., 2008).

Four developmental scales were developed which assessed the four core domains of child development: gross motor, fine motor, communication, and social skills. Each age appropriate developmental skill was scored as 2 if the child was reported to have completed the skill often, 1 if the skill had been completed only once or twice and 0 if the child had not yet completed the skill. Item scores were summed to create continuous scores for each developmental domain. Fine motor skills, gross motor and social skills were assessed using age appropriate questions at four times points (at 6, 18, 30, and 42 months), communication was assessed only twice (at 6 and 18 months). The attrition rate over time for each developmental skill ranged from 27 to 34%, and only children who completed assessments at all time points were included in the analysis. Items such as ‘my baby looks at older people’s faces,’ ‘he puts his hands together,’ ‘when a bell rings he moves or makes a noise,’ and ‘in a sitting position he can keep his head steady’ reflected social, fine, communication, and gross motor skills at 6 months, respectively. All analyses were adjusted for the actual age and performance of the child when the first questionnaire was completed. These adjustments minimize and take into consideration the concept of regression to the mean over time.

### Covariates

**Table 1** shows the frequencies and statistical significance of socio-demographic characteristics of the sample by PEs group. Child’s birth weight was obtained from hospital birth records and information on child’s gender and ethnicity (Caucasian versus non-Caucasian), maternal marital status (never married, married, separated/divorced), home ownership (owned/mortgage, rent), residential status (urban versus rural), occupational and educational qualifications (UK classification dichotomised as manual and non-manual), and family history of psychiatric problems were obtained from questionnaires during pregnancy. Child’s total IQ score was assessed using the Wechsler Intelligence Scale for Children at mean age 8 years. These variables are considered as potential confounders associated with developmental delays and/or PEs [e.g., IQ test performance and PEs (Horwood et al., 2008)]. However, adjusting results for all these covariates would have led to considerable loss of sample size due to missing data. Therefore, in this study, we presented adjusted results only for the variables that altered the unadjusted OR; these included child’s gender, total IQ, maternal residence, marital and educational status.

**Table 1:** Frequencies and statistical significance of socio-demographic characteristics of the sample by PEs groups (not present *N* = 5862; suspected or definite *N* = 928).

		Psychotic experiences Frequencies (%)		Binary logistic regression: subgroup difference^∗^	Overall cross tabulation
		Not present	Suspected or definite	OR (95% CI)	*p*-Values
**Child characteristics**					
Gender	Female vs. male	2956 (50.4%)	503 (54.2%)	1.16 (1.01–1.34)	<0.05
Ethnicity	Non-Caucasian vs. Caucasian	205 (3.9%)	37 (4.4%)	1.15 (0.81–1.65)	0.43
Birth weight (Kg) Mean (*SD*)		3.42 (0.54)	3.38 (0.57)	0.88 (0.78–1.00)	0.05
Total IQ – WISC (age 8) Mean (*SD*)		105.63 (16.71)	102.85 (17.11)	0.99 (0.98–0.99)	<0.001
**Parental characteristics**					
Urban/rural residence	Rural vs. urban	294 (5.5%)	47 (5.6%)	1.02 (0.74–1.40)	0.91
Family history of schizophrenia	Yes vs. no	38 (0.8%)	6 (0.8%)	1.01 (0.42–2.39)	0.99
Family history of depression	Yes vs. no	1267 (25.9%)	202 (26.3%)	1.02 (0.86–1.21)	0.83
**Maternal characteristics**					
Residential status	Rent vs. owned (mortgage)	857 (15.9%)	174 (20.5%)	1.37 (1.14–1.64)	<0.001
Marital status	Never married	730 (13.4%)	150 (17.7%)	Reference group (1)	
	Married	4465 (82.1%)	640 (75.5%)	0.71 (0.57–0.85)	
	Separated, divorced	242 (4.5%)	58 (6.8%)	1.17 (0.83–1.63)	<0.001
Highest education (UK)	<O level vs. ≥O level	901 (19.3%)	123 (21.3%)	1.13 (0.92–1.40)	0.25
Ethnic background	Non-Caucasian vs. Caucasian	100 (1.9%)	15 (1.8%)	0.95 (0.55–1.64)	0.85
Occupational status (UK)	Manual vs. non-manual	2323 (56.7%)	277 (56.0%)	0.97 (0.80–1.17)	0.75
History of schizophrenia	Yes vs. no	9 (0.3%)	1 (0.2%)	0.73 (0.09–5.78)	0.75
History of depression	Yes vs. no	979 (20.0%)	162 (21.1%)	1.15 (0.92–1.43)	0.49
**Paternal characteristics**					
Ethnic background	Non-Caucasian vs. Caucasian	150 (2.8%)	31 (3.7%)	1.33 (0.89–1.97)	0.16
Occupational status (UK)	Manual vs. non-manual	2096 (48.4%)	286 (54.2%)	1.26 (1.05–1.51)	<0.05
History of schizophrenia	Yes vs. no	20 (0.4%)	4 (0.5%)	0.82 (0.19–3.62)	0.67
History of depression	Yes vs. no	220 (6.5%)	37 (7.0%)	1.10 (0.76–1.57)	0.65

*PEs, psychotic experiences; WISC, Wechsler Intelligence Scale for Children; OR, odds ratio; 95% CI, confidence interval; ^∗^comparison between definite and suspected group with not present as reference category*.

### Data Manipulation and Statistical Analyses

All raw DDST – II scores were standardized to a mean of 0 and standard deviation of 1 (z-scores). Descriptive statistics were used to describe performance in developmental skills at 6, 18, 30, and 42 months by PEs grouping. Binary and multinomial logistic regression was used to examine the relationship between measurements of developmental skills and likelihood of reporting PEs.

In the present study, we used quantitative techniques uniquely suited for modeling and detecting developmental change over time. This study deployed a latent class growth analysis (LCGA) (mixture modeling approach) using Mplus (Muthén and Muthén, 2012) to derive trajectories of change in developmental skills acquisition during the first 4 years of life. Adjusting for age, trajectories of change in fine and gross motor and social skills were derived. Trajectory classes are empirically defined groups based on the longitudinal trends present in the data (Nagin, 2005).

The optimum number of classes or trajectories was determined using statistical fit indices criteria [highest entropy value (entropy ranges from 0.00 to 1.00, with higher values indicating more accurate classification), lowest Bayesian Information Criteria (BIC), non-significant Lo-Mendell-Rubin Likelihood Ratio Test (LMR-LRT) comparing the current model with a model with one less class]. These criteria have been shown to be consistent indicators of latent classes (Nylund et al., 2007). The general practice of LCGA is to test the fit of a two-class model and systematically increase the number of classes until adding more classes is no longer warranted (non-significant LMR-LRT) (Tein et al., 2013).

Communication skills were only measured at two time points (6 and 18 months), therefore an alternative method was used to examine prediction of PEs from trajectories of performance over time including consistently low vs. decline in communication skills z-scores. Principal component analysis (PCA) was used as a means of transforming the original communication variables to derive two uncorrelated (orthogonal) scores in order to avoid collinearity (Massy, 1965). A detailed explanation of this statistical technique is provided elsewhere (Hameed et al., 2013), and has been used in another study (Niarchou et al., 2013). Two fixed principal components with orthogonal (varimax) rotation for total communication score at 6 and 18 months were generated and saved as standardized regression scores. PCA yielded a first (eigenvalue = 1.26, percentage of variance = 63.14) and a second component (eigenvalue = 0.74, percentage of variance = 36.86) reflecting average and change score between the two time points respectively. These two standardized components were used as continuous predictors in subsequent logistic regression models to assess their association with PEs with and without adjustments for potential confounding variables.

## Results

### Participants and Missing Data

A total of 6790 (44.6%) children from the ALSPAC cohort (*N* = 15211) completed the PLIKS semi-structured interview. However, in this study, only 5345 (35.1%) children had complete data related to early developmental domains and PLIKS in early adolescence. Descriptive and inferential statistics regarding the developmental trajectories of children, who were and were not assessed with the PLIKS interview (Supplementary Table S1), reveal no major differences in the developmental domains (except social skills). Therefore, this study reports the results only for children who completed the PLIKS interview and also had complete data related to early childhood developmental domains.

### Sample Characteristics

Child demographic characteristics and intellectual profile were examined between PEs ‘not present’ versus PEs ‘suspected or definitely present’ group. These are reported in **Table 1**. Those with suspected/definite PEs were more likely to be female, have a lower IQ, and have parents who lived in rented accommodation, were unmarried, and had a manual occupational status; these domains represent proxy measures of social class according to UK classifications.

### Latent Class Growth Analysis

Results from model selection, proportion of individuals in each class and average latent class probabilities are shown in Supplementary Tables S2a,b respectively. The derived trajectory classes are based on the longitudinal trends – in terms of initial performance level and rate of change – present in the data (Nagin, 2005). The analyses were adjusted for age at initial assessment.

#### Fine Motor Skills

**FIGURE 1:**
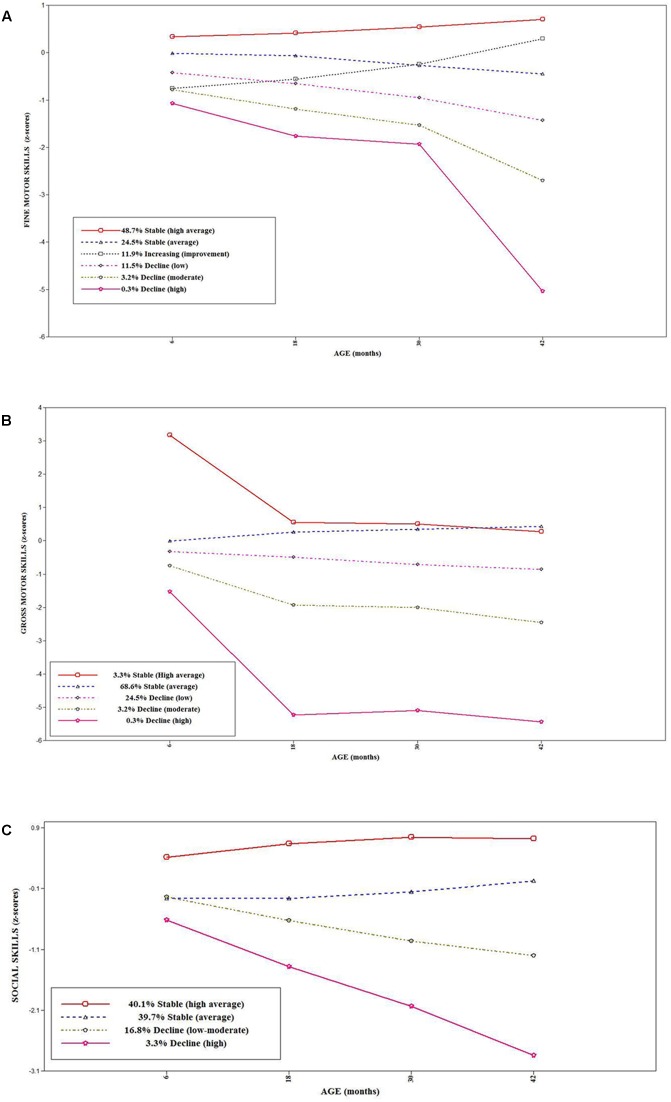
**(A)** Trajectories of fine motor skills over time. **(B)** Trajectories of gross motor skills over time. **(C)** Trajectories of social skills over time.

Based on the lowest BIC score, acceptable entropy, and a non-significant LMR-LRT test, a six class model provided the optimal solution for fine motor skills. **Figure 1A** shows that the largest class included those children with stable high average fine motor z-scores over time (48.7%). The second largest class included children with stable average scores over time (24.5%). A proportion of children had an improving performance over time (11.9%). Three classes were derived which represented a declining pattern of fine motor skills z-scores, relative to peers; low (11.5%), moderate (3.2%), and high (0.3%) declining z-scores over time.

Consistent with the research hypothesis, the stable average, high average, and improvement classes were grouped together reflecting “stable” performance. Similarly, the declining classes were classified as one group. This categorization is consistent with the research hypothesis that a group of children with declining versus stable pattern of skills acquisition is predictive of PEs. Furthermore, since only a small percentage of participants had a pattern of declining performance, grouping them as one class reflecting decline, would increase statistical power.

#### Gross Motor Skills

A five class model provided the optimal solution. **Figure 1B** shows that the largest class included those with stable average scores (68.6%). However, only 3.3% had a high average scores (while with an initial decline, this class remained above average). A considerable proportion of children were classified as having a low (24.5%) declining pattern of performance, relative to peers. Furthermore, two small proportions were classified as having moderate (3.2%) and high (0.3%) declining patterns of gross motor skills over time. The groups with high and stable average scores over time were grouped together and termed stable class. Those with a low, moderate, and high declining pattern of performance were grouped together and classified as the declining group.

#### Social Skills

A four class categorization provided the best fitting model. **Figure 1C** shows that approximately 80% of children were classified as having high average or average social z-scores over time. These were grouped to form a stable class. The two other classes included children with low-moderate (16.8%) and high (3.3%) declining social z-scores over time, relative to their peers. These were grouped together and classified as declining class.

The derived classes (declining and stable) were used as categorical predictors of PEs using logistic regression with and without adjustment for potential confounding variables.

### Associations between Early Childhood Developmental Trajectories and Early Adolescent PEs

**Table 2** shows the frequencies and percentages of fine, gross motor, social, and communication skills z-score trajectories (stable and decline) by PEs groupings. Furthermore **Table 3** shows the odds ratios for decline versus stable in early childhood developmental domains and PEs (not present as reference category).

**Table 2:** Frequencies and percentages for early childhood developmental trajectories and psychotic experiences in early adolescence.

Denver developmental skills (trajectories over time) [z-scores]	Psychotic experiences frequencies (percentages)			
	Not present (*n* = 4636)	Suspected (*n* = 420)	Definite (*n* = 289)	Suspected and definite (*n* = 709)
**Fine motor skills (z-scores)**				
Stable	3981 (85.9%)	360 (85.7%)	239 (82.7%)	599 (84.5%)
Decline	655 (14.1%)	60 (14.3%)	50 (17.3%)	110 (15.5%)
**Gross motor skills**				
Stable	3438 (74.2%)	321 (76.4%)	218 (75.4%)	539 (76.0%)
Decline	1198 (25.8%)	99 (23.6%)	71 (24.6%)	170 (24.0%)
**Social skills**				
Stable	3754 (81.0%)	323 (76.9%)	240 (83.0%)	563 (79.4%)
Decline	882 (19.0%)	97 (23.1%)	49 (17.0%)	146 (20.6%)
**Communication skills**				
Stable	3866 (83.4%)	342 (81.5%)	237 (82.0%)	579 (81.6%)
Decline	770 (16.6%)	78 (18.5%)	52 (18.1%)	130 (18.4%)

**Table 3:** Odds ratios for early childhood developmental trajectories and psychotic experiences (not present as reference category, *n* = 4636).

Denver developmental skills (trajectories over time) [z-scores]	Psychotic experiences OR (95% confidence interval)				
	Suspected^A^		Definite^A^		Suspected and definite^B^	
	OR	Adjusted OR	OR	Adjusted OR	OR	Adjusted OR
**Fine motor skills (z-scores)**						
Decline vs. Stable	1.01 (0.76–1.35)	1.08 (0.77–1.53)	1.27 (0.93–1.74)	1.36 (0.93–1.99)	1.12 (0.90–1.40)	1.19 (0.92–1.55)
*p*-values	0.929	0.942	0.136	0.099	0.150	0.305
**Gross motor skills**						
Decline vs. Stable	0.89 (0.70–1.12)	0.96 (0.73–1.27)	0.94 (0.71)–1.23)	0.85 (0.60–1.19)	0.91 (0.75–1.09)	0.91 (0.74–1.14)
*p*-values	0.308	0.344	0.631	0.539	0.290	0.276
**Social skills**						
Decline vs. Stable	1.45 (1.13–1.89)	1.28 (1.10–1.92)	0.87 (0.63–1.19)	0.84 (0.57–1.24)	1.10 (0.91–1.34)	1.19 (0.95–1.50)
*p*-values	0.004	0.044	0.384	0.395	0.324	0.094
**Communication skills**						
**Principal component (1)**						
Average	0.96 (0.87–1.06)	0.96 (0.86–1.08)	1.06 (0.94–1.19)	1.02 (0.89–1.17)	1.0 (0.92–1.09)	0.99 (0.90–1.08)
*p*-values	0.401	0.485	0.327	0.740	0.930	0.747
**Principal component (2)**						
Change (decline)	1.12 (1.01–1.23)	1.11 (0.99–1.24)	1.16 (1.03–1.30)	1.13 (0.99–1.29)	1.13 (1.04–1.23)	1.12 (1.03–1.22)
*p*-values	0.031	0.058	0.012	0.58	0.005	0.010

**^A^***Multinomial logistic regression; **^B^**binary logistic regression; OR, odds ratio; Adjusted OR, odds ratio adjusted for child’s gender, total IQ, maternal residential, marital, and educational status*.

#### Fine Motor Skills

The results found that 17.3% of children with definite PEs had declining fine motor z-score compared to 14.3% of the suspected, and 14.1% of those with no PEs. However, there were no evidence of an association between fine motor skills and PEs. **Tables 2**, **3** show the descriptive statistics and the relevant odds ratios.

#### Gross Motor Skills

There was no evidence of an association between declining gross motor skills z-scores and PEs in early adolescence. Indeed, 170 (24.0%) of those with ‘suspected or definite’ PEs were classified as having a declining trend of gross motor z-scores in comparison to those without PEs (*n* = 1198, 25.8%; adjusted OR = 0.91, 95% CI = 0.74–1.14).

#### Social Skills

Those with suspected or definite PEs had lower social z-scores at 42 months (*M* = -0.08, *SD* = 1.02) in comparison to those without PEs (*M* = 0.01, *SD* = 0.99), with an increase of 1 standard deviation in social skills z-scores associated with decreased risk of reporting PEs (OR = 0.92, 95% CI = 0.85–0.99) (see Supplementary Tables S3a,b). However, in terms of trajectories of social skills z-scores, only those with ‘suspected’ PEs were more likely to have a declining pattern of social skills over time (adjusted OR = 1.28, 95% CI = 1.10–1.92).

#### Communication Skills

Those children with ‘suspected and definite’ PEs were found to have higher communication z-scores at 6 months than those where PEs were not present (OR = 1.14, 95% CI = 1.05–1.23). Compared to those without PEs, those with ‘suspected or definite’ PEs had higher communication scores at both 6 and 18 months (Supplementary Table S3a). However, there were no differences in communication skills measured at 18 months.

More specifically, in terms of communication skills over time, results showed no evidence that a consistently low average communication skills is associated with increased risk of ‘suspected or definite’ PEs (adjusted OR = 0.99, 95% CI = 0.90–1.08). However, there was evidence of an association between changing (decline) communication skills z-scores and PEs (adjusted OR = 1.12, 95% CI = 1.03–1.22).

### Gender Specific Effects

The results found that males had significantly higher rates of declining trend of fine (29.8%), gross motor (23.6%), social (33.0%) and communication skills (20.7%), than females (19.3, 18.6, 25.2, and 15.7% respectively). However, there was no evidence that associations between childhood developmental skills and PEs differed according to gender. The exception was only that declining pattern of fine motor skills was associated with odds of PEs in males compared to females (interaction OR = 1.47, 95% CI = 1.09–1.97, *p* = 0.012).

## Discussion

This study contributed to our understanding of the developmental pathway toward (PEs) by examining the association between trajectories of early developmental skills over early childhood and PEs in early adolescence. We observed a small increase in likelihood of PEs in children with a decline in social and communication skills. In contrast to previous research we found no strong association between declining fine or gross motor development and later PEs. Overall the magnitude of the association with predictive values were weak and not strongly supporting study’s hypotheses.

In terms of previous research, a study in adolescence had found that a pattern of delays in childhood from 3 years onward in motor and in language development were associated with PEs (Cannon et al., 2002). While Cannon et al.’s (2002) study examined children’s motor skills between the ages of 3 and 9 years, our study examined a considerably earlier period of child development (between 6 and 42 months). In contrast to Cannon’s findings, our examination of declining patterns of motor performance (fine and gross motor skills) over time was not associated with PEs. It is possible that a pattern of delay in acquiring motor skills later during the child’s development, and not as early as 6–42 months, may be associated with PEs as demands for complex motor functioning increase with age. Alternatively, the assessment of early childhood developmental skills used in this study (adapted parental report of DDST – II), though moderately correlated with the Griffith’s (*r* = 0.54), may not be sufficiently sensitive to detect differences in developmental domains. Furthermore, it is not clear as to (a) why this correlation is not higher, as it is based on an observer report? and (b) to what is the remaining variance attributable?

Overall, while there was some evidence for the association of social and communication skills with PEs, the findings related to motor skills were not consistent with previous birth cohort and high-risk prospective studies that have investigated early childhood developmental factors and schizophrenia-spectrum disorders in adulthood (Walker et al., 1994; Owens and Johnstone, 2006; Welham et al., 2009b). The inconsistency of findings with previous studies may imply that PEs in adolescence may, in the main, not emerge as a consequence of the same early neurodevelopmental aetiologies underlying schizophrenia-spectrum disorders. Alternatively, major neurodevelopmental changes happening later, during adolescence may considerably add toward the expression of affective dysregulation manifested in PEs and/or depressive symptoms (Fonseca-Pedrero et al., 2011). However, these alternative explanations warrant further research.

In addition, results differed somewhat according to whether PEs were defined as definite or suspected. Generally the stronger associations were for the suspected category, though confidence intervals overlapped substantially and no evidence of a difference between suspected and definite. Furthermore, numbers in the definite category are quite small, leading to reduced power in analysis. In addition, it is also possible that subtle differences in early childhood developmental skills acquisition (e.g., fine, gross motor, social, and communication skills) become more pronounced over a longer timeframe and that association would be stronger if the measures are repeated for a longer duration across childhood and adolescence. However, such proposition is subject to further research.

This study used a person-oriented approach to analysis, which assumes that there are meaningful subgroups of individuals who have common pathways of development (Sterba and Bauer, 2010). This form of analysis allows a more robust test of a developmental model of liability to PEs, based on the assumption that developmental deficits become more pronounced across time. While the majority of previous studies have focused on lower average level of functioning in a group at a given time point in development as predictor of PEs and/or schizophrenia-spectrum disorders (Welham et al., 2009a), the current person-oriented approach suggested that it is the pattern of development for the individual child across time which is likely to be more predictive of adolescent PEs, although the findings only partially supported this argument.

The main strengths of this study were the large sample size, the prospective design, repeated recordings of the developmental variables, and the quality control in place in the measurement of PEs (with a moderate inter-rater reliability, *k* = 0.72) (Horwood et al., 2008). While this is considered as less than optimal inter-rater reliability estimate, the semi-structured interview used to assess PEs is likely to have resulted in less measurement error. Trained interviewers conducted the PEs semi-structured interviews, and these are likely to be more valid for assessing PEs than structured instruments or self-report, as they allow cross-questioning of the kind used in clinical practice.

Due to the richness of the cohort data, this study had access to a comprehensive list of potential covariates. Nevertheless, the results of this study need to be interpreted with caution owing to several limitations. There may be other confounders related to early development and/or emergence of PEs, which perhaps should have been included, such as parental use of alcohol and other drugs during pregnancy. Data on risk of early child abuse and neglect (Read et al., 2005), exposure to traumatic experiences, or data on children’s social care (children with a slower development could have received early social care), which may potentially be related to risk of emergence of PEs, was not considered. Moreover, this study did not consider other possible traumatic events and early adverse experiences such as traumatic death of an immediate family member (Morgan et al., 2007). Furthermore, moderating and mediating variables, or the possibility of a third variable mutually influencing early childhood developmental skills and PEs in early adolescence warrant further research.

In terms of the completion rate, more than 50% of the original total participants in this study did not complete the PLIKS interviews; these were conducted at ALSPAC clinics which considerably add to reduction in response rate. Furthermore, the data for this study relied on parental report to score their child’s developmental profiles; and may have been subject to measurement error, either under or over scoring (Emond et al., 2005). Also, while beyond the scope of this study, this paper only focused on PEs as an outcome criteria, it is highly recommended that future research examine the pseudo-specificity of trajectories of early developmental skills related to PEs over psychopathology in general. Additionally, not all adolescents reporting PEs would develop psychosis or any other form of psychopathology later in life. While, this is critical to understand the pseudo-specificity of the findings, it is beyond the scope and outside the aims of this study.

In this study, declining patterns of early developmental domains of social and communication skills ‘z-scores’ (measured by parental report) were weakly associated with PEs, and largely not supporting the study’s hypotheses. It is unknown whether such a pattern of increased risk of PEs with declining performance occurs across other domains of psychological development and/or is accompanied by a progressive regression in development. Additional research is needed using advanced techniques and assessing deterioration in developmental abilities over time. The extent and magnitude to which subtle differences in early childhood developmental skills persist throughout adolescence and adulthood, and are predictive of PEs and adult onset of schizophrenia-spectrum disorders remains unknown. Therefore, future research may also examine the association of subtle developmental differences and PEs in older children and adolescents, since these may only become evident later in development.

Furthermore, within the context of the wide range of other risk factors identified for schizophrenia-spectrum disorders (Owens and Johnstone, 2006; Welham et al., 2009a), it seems unlikely that the developmental domains examined in this study are key causal factors in the pathogenesis of PEs and/or psychosis in general. Instead, consistent with neurodevelopmental models of the casual mechanism leading to psychosis, subtle developmental differences are more likely to reflect a broader pathological processes (Rapoport et al., 2012). In addition, the indication that an early insult in development plays an important role in the origin of psychotic conditions require further investigation to identify specific likely causes. This would be a key finding in the field of developmental psychopathology.

On a broader scale, if PEs in early adolescence are a risk factor for adult onset psychotic conditions, they could contribute toward efforts of early identification of those with greater risk of developing a future psychotic disorder. However, the evidence for continuity of PEs from early adolescence to adulthood is not specific or highly predictive of psychotic disorders in adulthood (Zammit et al., 2013). Further, the examined childhood developmental factors may not be specific to only PEs, but also predictive of other psychopathologies. Nevertheless, the findings of this study, along with the growing body of work is applicable to the development of early detection strategies and preventative interventions. However, while prevention of the spectrum of psychotic conditions is a “desirable aim,” this is not “possible without accurate identification of those at risk before the onset of illness” (Sullivan et al., 2013b, p. 1053). This field will require considerable further translational research directed toward accurate identification of risk factors, which could potentially be used to define an intervention target group consisting of children with a highly elevated risk of developing schizophrenia-spectrum disorders.

## Conclusion

This study recommends that clinicians and researchers should focus on the development of risk assessment strategies for children who may have an elevated risk of developing PEs, and arguably schizophrenia-spectrum disorders. This study was one of many that focussed on early childhood developmental indicators of PEs in early adolescence. However, the association between PEs in early adolescence and the incidence of schizophrenia-spectrum disorders in adulthood should further be investigated in mature cohorts.

## Author Contributions

MH wrote the first draft of the manuscript. Subsequent drafts of the manuscript were edited by all authors, who contributed to and approved the final manuscript. All authors developed the concepts and contributed to the analytical plan for this study.

## Conflict of Interest Statement

The authors declare that the research was conducted in the absence of any commercial or financial relationships that could be construed as a potential conflict of interest.
