# Supplementary material for: Trajectories of Early Childhood Developmental Skills and Early Adolescent Psychotic Experiences: Findings from the ALSPAC UK Birth Cohort
Source: Front Psychol. 2018 Jan 9;8:2314. doi: 10.3389/fpsyg.2017.02314 (PMC5767306; doi:10.3389/fpsyg.2017.02314)
Supplement: Supplementary file 1 [file Table_1.docx]

Table S1. Descriptive and inferential statistics for trajectories of Denver Developmental Screening Test- II domains by PLIKS completion status

|  | **Psychotic experiences**  **PLIKS Interview** | |  |
| --- | --- | --- | --- |
| **Denver Developmental Skills (trajectories over time)** | **Completed (n = 5339)** | **Not completed (1750)** | **Statistics** |
| **Fine Motor Skills** | **N (%)** | **N (%)** | ***p.* values** |
| Stable | 4575 (85.7%) | 1500 (85.7%) |  |
| Decline | 764 (14.3%) | 250 (14.3%) | 0.980 |
| **Gross Motor Skills** |  |  |  |
| Stable | 3973 (74.4%) | 1288 (73.6%) |  |
| Decline | 1366 (25.6%) | 462 (26.4%) | 0.499 |
| **Social Skills** |  |  |  |
| Stable | 4311 (80.7%) | 1455 (83.1%) |  |
| Decline | 1028 (19.3%) | 295 (16.9%) | 0.025 |
| **Communication skills** |  |  |  |
| Average (Mean [z-score], SD) | -.01 (.99) | .03 (1.02) | 0.249 |
| Change (Mean [z-score], SD) | -.01 (.99) | .04 (1.04) | 0.073 |

**Notes: PLIKS =** Psychotic-Like Symptoms Semi-Structured Interview;
