# Supplementary material for: Trajectories of Early Childhood Developmental Skills and Early Adolescent Psychotic Experiences: Findings from the ALSPAC UK Birth Cohort
Source: Front Psychol. 2018 Jan 9;8:2314. doi: 10.3389/fpsyg.2017.02314 (PMC5767306; doi:10.3389/fpsyg.2017.02314)
Supplement: Supplementary file 2 [file Table_2.docx]

Table S2.a. Model selection results from latent class growth analysis (mixture) of Denver Developmental Screening Test - II

| **DDST – II: Fine Motor Skills** | | | | | |
| --- | --- | --- | --- | --- | --- |
|  | | | | **LMR-LRT** | |
| **GMM classes** | **Likelihood** | **BIC** | **Entropy** | **^2^log Likelihood** | ***p.* value** |
| 1 | -37334.13 | 74721.47 | ----- | ----- | ----- |
| 2 | -35452.02 | 70983.84 | .785 | 3764.24 | 0.0000 |
| 3 | -35020.61 | 70147.63 | .762 | 862.80 | 0.0003 |
| 4 | -34838.94 | 69810.88 | .784 | 363.36 | 0.0290 |
| 5 | -34732.49 | 69624.59 | .798 | 212.89 | 0.0446 |
| 6 | -34632.62 | 69451.45 | .735 | 198.08 | 0.0484 |
| 7 | -34542.12 | 69220.79 | .705 | 180.989 | 0.0769 |
| **DDST – II: Gross Motor Skills** | | | | | |
|  | | | | **LMR-LRT** | |
| **GMM classes** | **Likelihood** | **BIC** | **Entropy** | **^2^log Likelihood** | ***p.* value** |
| 1 | -37807.11 | 75667.43 | ----- | ----- | ----- |
| 2 | -36015.42 | 72110.65 | .840 | 3583.38 | 0.0000 |
| 3 | -35417.12 | 70940.65 | .828 | 1196.60 | 0.0000 |
| 4 | -35179.31 | 70491.63 | .824 | 475.62 | 0.0046 |
| 5 | -34863.96 | 69887.53 | .837 | 437.21 | 0.0038 |
| 6 | -34747.61 | 69681.44 | .844 | 232.68 | 0.1105 |
| **DDST – II: Social Skills** | | | | | |
|  | | | | **LMR-LRT** | |
| **GMM classes** | **Likelihood** | **BIC** | **Entropy** | **^2^log Likelihood** | ***p.* value** |
| 1 | -37462.36 | 74977.93 | ----- | ----- | ----- |
| 2 | -35312.68 | 70705.16 | .752 | 4299.37 | 0.0000 |
| 3 | -34542.95 | 69192.30 | .724 | 1539.46 | 0.0000 |
| 4 | -34273.69 | 68680.39 | .696 | 538.51 | 0.0000 |
| 5 | -34188.69 | 68537.00 | .680 | 169.99 | 0.2010 |

**Notes:** **DDST – II** = Denver Developmental Screening Test – II; **GMM** = Growth Mixture Model; **BIC** = Bayesian Information Criteria; **LMR-LRT** = Lo-Mendell-Rubin Likelihood Ratio Test; **Entropy** reported to 3 decimal places; ***p. values*** reported to 4 decimal places;

Table S2.b. Proportion of individuals in each class and average latent class probabilities for most likely latent class membership (row) by latent class (column)

|  | **Latent Class Analysis: Growth Mixture Model** | | | | | | | | | | | |
| --- | --- | --- | --- | --- | --- | --- | --- | --- | --- | --- | --- | --- |
| **Denver Developmental Skills** | **Number of individuals in class (proportion)** | | | | | | **Average latent class probabilities** | | | | | |
|  | **1** | **2** | **3** | **4** | **5** | **6** | **1** | **2** | **3** | **4** | **5** | **6** |
| **Fine motor skills** |  |  |  |  |  |  |  |  |  |  |  |  |
|  | 7095 (1.00) | ----- | ----- | ----- | ----- | ----- | 1.00 | ----- | ----- | ----- | ----- | ----- |
|  | 1401 (.20) | 5694 (.80) | ----- | ----- | ----- | ----- | .88 | .95 | ----- | ----- | ----- | ----- |
|  | 2003 (.28) | 4758 (.67) | 334 (.05) | ----- | ----- | ----- | .83 | .92 | .87 | ----- | ----- | ----- |
|  | 628 (.09) | 69 (.01) | 4273 (.60) | 2125 (.30) | ----- | ----- | .86 | .88 | .91 | .81 | ----- | ----- |
|  | 238 (.03) | 837 (.12) | 18 (.002) | 3898 (.55) | 2104 (.30) | ----- | .86 | .83 | .98 | .90 | .80 | ----- |
|  | 1788 (.25) | 577 (.10) | 207 (.03) | 3721 (.49) | 19 (.003) | 783 (.11) | .74 | .63 | .88 | .86 | .96 | .81 |
| **Gross motor skills** |  |  |  |  |  |  |  |  |  |  |  |  |
|  | 7097 (1.00) | ----- | ----- | ----- | ----- | ----- | 1.00 | ----- | ----- | ----- | ----- | ----- |
|  | 925 (.13) | 6172 (.87) | ----- | ----- | ----- | ----- | .87 | .97 | ----- | ----- | ----- | ----- |
|  | 102 (.01) | 5529 (.78) | 1466 (.21) | ----- | ----- | ----- | .92 | .94 | .85 | ----- | ----- | ----- |
|  | 18 (.002) | 5164 (.73) | 1690 (.24) | 225 (.03) | ----- | ----- | .97 | .92 | .83 | .87 | ----- | ----- |
|  | 1601 (.24) | 18 (.003) | 209 (.03) | 5058 (.69) | 211 (.03) | ----- | .82 | .98 | .92 | .91 | .87 | ----- |
| **Social Skills** |  |  |  |  |  |  |  |  |  |  |  |  |
|  | 7098 (1.00) | ----- | ----- | ----- | ----- | ----- | 1.00 | ----- | ----- | ----- | ----- | ----- |
|  | 1763 (.25) | 5335 (.75) | ----- | ----- | ----- | ----- | .88 | .94 | ----- | ----- | ----- | ----- |
|  | 2819 (.40) | 3736 (.53) | 543 (.07) | ----- | ----- | ----- | .84 | .89 | .88 | ----- | ----- | ----- |
|  | 1101 (.16) | 2908 (.40) | 223 (.03) | 2866 (.39) | ----- | ----- | .83 | .77 | .89 | .85 | ----- | ----- |
