# Supplementary material for: Trajectories of Early Childhood Developmental Skills and Early Adolescent Psychotic Experiences: Findings from the ALSPAC UK Birth Cohort
Source: Front Psychol. 2018 Jan 9;8:2314. doi: 10.3389/fpsyg.2017.02314 (PMC5767306; doi:10.3389/fpsyg.2017.02314)
Supplement: Supplementary file 3 [file Table_3.docx]

Table S3.a. Descriptive statistics for standardised z scores of Denver Developmental Screening Test- II domains and psychotic experiences

| **Developmental Domain of functioning** |  | **Psychotic Experiences**  **Mean (standard deviation)** | | | |
| --- | --- | --- | --- | --- | --- |
| Fine motor skills (time points) | **Attrition rate at each time point (%)** | **Not present**  **(n = 4228)** | **Suspected**  **(n = 381)** | **Definite**  **(n = 258)** | **Suspected and definite (n = 639)** |
| 6 (months) | 31 | -.02 (1.00) | .05 (.99) | .06 (1.04) | .05 (1.01) |
| 18 | 27 | .01 (.98) | -.03 (1.15) | -.08 (1.04) | -.05 (1.11) |
| 30 | 32 | .03 (.99) | -.06 (.97) | .04 (1.07) | -.02 (1.02) |
| 42 | 34 | .02 (.99) | -.01 (.98) | -.08 (1.11) | -.04 (1.04) |
| Gross motor Skills |  |  |  |  |  |
| 6 (months) | 27 | .00 (.99) | .03 (1.02) | -.02 (.91) | .01 (.98) |
| 18 | 27 | -.02 (.97) | -.05 (1.17) | .04 (1.05) | -.01 (1.12) |
| 30 | 32 | -.02 (.99) | -.06 (1.08) | .00 (1.05) | -.04 (1.07) |
| 42 | 34 | -.02 (1.00) | -.04 (1.05) | -.02 (1.04) | -.03 (1.04) |
| Social skills |  |  |  |  |  |
| 6 (months) | 30 | .00 (1.00) | .03 (1.00) | .01 (1.02) | .02 (1.01) |
| 18 | 27 | .00 (.98) | .00 (1.02) | .03 (1.03) | .01 (1.02) |
| 30 | 32 | .00 (.99) | -.08 (.98) | .05 (1.00) | -.02 (.99) |
| 42 | 34 | .01 (.99) | -.11 (1.03) | -.03 (1.00) | -.08 (1.02) |
| Communication skills |  |  |  |  |  |
| 6 (months) | 29 | -.04 (.98) | .06 (.95) | .13 (.99) | .09 (.97) |
| 18 | 27 | -.01 (.98) | -.01 (1.04) | .07 (1.08) | .02 (1.06) |

**Notes: n** = number of participants; **SD** = standard Deviation;

Table S3.b. Odds ratio for standardised z-scores of Denver Developmental Screening Test- II domains and psychotic experiences (not present as reference category)

| **Developmental Domain of functioning** | **Psychotic Experiences**  **Odds ratios (95% Confidence Interval)** | | |
| --- | --- | --- | --- |
| Fine motor skills (time points) | **Suspected ^a^** | **Definite ^a^** | **Suspected and definite ^b^** |
| 6 (months) | 1.07 (.96-1.19) | 1.09 (.96-1.23) | 1.08 (.99-1.17) |
| 18 | .96 (.87-1.07) | .92 (.82-1.03) | .95 (.87-1.02) |
| 30 | .92 (.83-1.02) | 1.01 (.89-1.15) | .96 (.88-1.04) |
| 42 | .98 (.88-1.09) | .91 (.81-1.02) | .95 (.88-1.03) |
| Gross motor Skills |  |  |  |
| 6 (months) | 1.03 (.93-1.14) | .98 (.87-1.12) | 1.01 (.93-1.10) |
| 18 | .97 (.88-1.07) | 1.07 (.94-1.21) | 1.01 (.93-1.10) |
| 30 | .96 (.87-1.07) | 1.02 (.90-1.16) | .99 (.91-1.07) |
| 42 | .98 (.88-1.08) | .99 (.88-1.13) | .99 (.91-1.07) |
| Social skills |  |  |  |
| 6 (months) | 1.03 (.93-1.15) | 1.01 (.89-1.14) | 1.02 (.94-1.11) |
| 18 | 1.00 (.90-1.11) | 1.03 (.91-1.17) | 1.01 (.93-1.10) |
| 30 | .92 (.83-1.03) | 1.06 (.94-1.20) | .98 (.90-1.06) |
| 42 | **.89 (.81-.99)** | .96 (.85-1.09) | **.92 (.85-.99)** |
| Communication skills |  |  |  |
| 6 (months) | **1.11 (1.00-1.22)** | **1.18 (1.05-1.33)** | **1.14 (1.05-1.23)** |
| 18 | 1.01 (.91-1.11) | 1.09 (.96-1.23) | 1.04 (.96-1.13) |

**Notes:** ^a^ = Multinomial logistic regression; ^b^ = Binary logistic regression; bolded odds ratios are statistically significant
